# Delirium Tremens

**Published:** 1874-05

**Authors:** 


Bellevue Hospital.—*Delirium Tremens.*—What this class of patients most need is food and rest. Generally, if rest can be secured, there will be no further trouble in the management of the patients, for they will return to taking food, and in a short time the immediate effects of their debauch have passed.

Rest is usually secured by the administration of either potass. bromid. in large doses, grs. xxx, every two hours perhaps, or chloral hydrat. grs. xx, every hour. It is necessary in using the latter remedy to be cautious with regard to the size of the first doses, until the strength of the preparation and the susceptibility of the patient is fully understood. Then it may, perhaps, be given in much larger doses than indicated. Occasionally even large doses will be of no avail.

A very common prescription employed is the following:

℞.—Potass. bromid, . . . . ʒ iiss.
Chloral hydrat, . . . . . ʒ iss.
Syr. aurantii,
Aquæ, . . . . . . . aa. ℥ij.
M.—S. Half fluid ounce, and repeated every hour if necessary to procure sleep.

